# Lamotrigine and levetiracetam exert a similar modulation of TMS‐evoked EEG potentials

**DOI:** 10.1111/epi.13599

**Published:** 2016-11-03

**Authors:** Isabella Premoli, Andrea Biondi, Sara Carlesso, Davide Rivolta, Mark P. Richardson

**Affiliations:** ^1^ Department of Basic and Clinical NeuroscienceInstitute of Psychiatry, Psychology and Neuroscience (IoPPN)King's College LondonLondonUnited Kingdom; ^2^ School of PsychologyUniversity of East London (UEL)LondonUnited Kingdom

**Keywords:** Electroencephalography, Epilepsy, Pharmaco‐TMS‐EEG, Antiepileptic drug, Transcranial magnetic stimulation

## Abstract

**Objective:**

Antiepileptic drug (AED) treatment failures may occur because there is insufficient drug in the brain or because of a lack of relevant therapeutic response. Until now it has not been possible to measure these factors. It has been recently shown that the combination of transcranial magnetic stimulation and electroencephalography (TMS‐EEG) can measure the effects of drugs in healthy volunteers. TMS‐evoked EEG potentials (TEPs) comprise a series of positive and negative deflections that can be specifically modulated by drugs with a well‐known mode of action targeting inhibitory neurotransmission. Therefore, we hypothesized that TMS‐EEG can detect effects of two widely used AEDs, lamotrigine and levetiracetam, in healthy volunteers.

**Methods:**

Fifteen healthy subjects participated in a pseudo‐randomized, placebo‐controlled, double‐blind, crossover design, using a single oral dose of lamotrigine (300 mg) and levetiracetam (3,000 mg). TEPs were recorded before and 120 min after drug intake, and the effects of drugs on the amplitudes of TEP components were statistically evaluated.

**Results:**

A nonparametric cluster‐based permutation analysis of TEP amplitudes showed that AEDs both increased the amplitude of the negative potential at 45 msec after stimulation (N45) and suppressed the positive peak at 180 msec (P180). This is the first demonstration of AED‐induced modulation of TMS‐EEG measures.

**Significance:**

Despite the different mechanism of action that lamotrigine and levetiracetam exert at the molecular level, both AEDs impact the TMS‐EEG response in a similar way. These TMS‐EEG fingerprints observed in healthy subjects are candidate predictive markers of treatment response in patients on monotherapy with lamotrigine and levetiracetam.

**Key Points**

- TMS‐EEG is a noninvasive in vivo method to measure the effects of drugs acting in the human brain
- Lamotrigine and levetiracetam modulate the TMS‐evoked EEG response to a similar extent irrespective of their molecular target
- These TMS‐EEG fingerprints may indicate candidate predictive markers of treatment response in patients on monotherapy with lamotrigine and levetiracetam

Antiepileptic drugs (AEDs) are widely used for the treatment of epilepsy and sometimes used to treat other conditions such as migraine and bipolar disorder. It is assumed that they act to reduce neural firing, epileptic synchronization, and seizure spread.^1^ Some AEDs suppress neural membrane excitability by blocking voltage‐dependent Na^+^/Ca^2+^ channels; others increase γ‐amino butyric acid (GABA)–mediated inhibitory neurotransmission, or antagonize excitatory glutamate neurotransmission.^1^ Despite the wide range of mechanisms of action, epileptic seizures are refractory to AEDs in 30% of cases.^2^ An inadequate response to AEDs may occur because the drug does not enter or remain in the brain, or because it does not exert a sufficient pharmacologic response. Until now, neither of these factors has been directly measureable, and the reason for treatment failure is therefore usually unknown. Thus, it would be an important step forward to directly measure the pharmacologic effect of AEDs in the human brain.

Transcranial magnetic stimulation combined with electromyography (TMS‐EMG) has been used to characterize the effect of AEDs in the brain and the relationship between these effects and therapeutic response in patients.^3^ For instance, the TMS parameter named resting motor threshold (RMT) was associated with ion channel conductivity as it was increased after the administration of AEDs targeting Na^+^ channels.^4^ Furthermore, an important clinical study in patients with new‐onset epilepsy showed an association between an increase of RMT values soon after commencement of first AED treatment and long‐term seizure control.^5^ However, these TMS‐EMG measures are not used clinically, and markers for AED responsiveness are still lacking.

To increase the power of TMS as a tool to directly investigate the brain, TMS has been combined with electroencephalography (TMS‐EEG).^6^ It has been shown recently that TMS‐EEG is a successful tool to directly measure the activity of drugs in the brain in healthy volunteers.^7^, ^8^ The complex EEG response to single‐pulse TMS stimulation is a sequence of positive and negative peaks at specific latencies (i.e., P25, N45, P70, N100, and P180), named TMS‐evoked EEG potentials (TEPs). TEPs have been analyzed before and after the administration of drugs with a well‐known mechanism of action on inhibitory GABAergic neurotransmission. Results showed that the N45 and N100 potentials are associated with GABA_A_ and GABA_B_ receptor–mediated inhibition, respectively.^7^

Recently, TMS‐EEG has been used to assess abnormal cortical excitability patterns in epilepsy populations. Patients with Unverricht‐Lundborg–type progressive myoclonus epilepsy showed an increase of the early positive waveform and suppression of late components.^9^ In contrast, patients with juvenile myoclonic epilepsy were found to have increased N100 and P180 amplitude after sleep deprivation.^10^ Finally, patients with epilepsy from periventricular nodular heterotopias showed abnormal TMS‐EEG activity at late latencies in line with findings from patients with focal epilepsy.^11^, ^12^ However, patients included in these studies were receiving AED treatment, which could per se alter the morphology of TEPs.

Here, we investigated TMS‐EEG effects of two of the most prescribed AEDs, lamotrigine and levetiracetam,^13^ in healthy volunteers. The TEP signatures of AED activity in normal subjects may provide candidate biomarkers of treatment response, which should be validated in future clinical studies. In addition, we believe that our findings will add new insight on the neurophysiologic origin of TEPs, which could be used in the future to identify the mechanism of action of newly discovered drugs.

## Materials and Methods

### Subjects

Fifteen male subjects aged 19–34 years (mean age ± standard deviation [SD] 25.2 ± 4.62 years) were recruited to the study from a local research volunteer database. One subject only was not able to complete the TMS‐EEG recording after the intake of lamotrigine; therefore, the total number of subjects for this condition is fourteen. Female participants were excluded due to evidence of menstrual cycle–related effects on cortical excitability, which could introduce a confound.^14^ All the subject were right‐handed according to the Edinburgh Handedness Inventory (laterality score ≥ 75%).^15^ Before the experiment, subjects underwent a physical examination and checked for any contraindications to TMS. Exclusion criteria included the use of central nervous system (CNS) active drug, abuse of any kind of drugs (nicotine/alcohol included), contraindications to the study medications (levetiracetam/lamotrigine) and a history of psychiatric or neurologic disease. The experimental protocol was approved by King's College London Research Ethics Committee, and all the participants gave their written informed consent before the experiment.

### Experimental design

**Figure 1:**
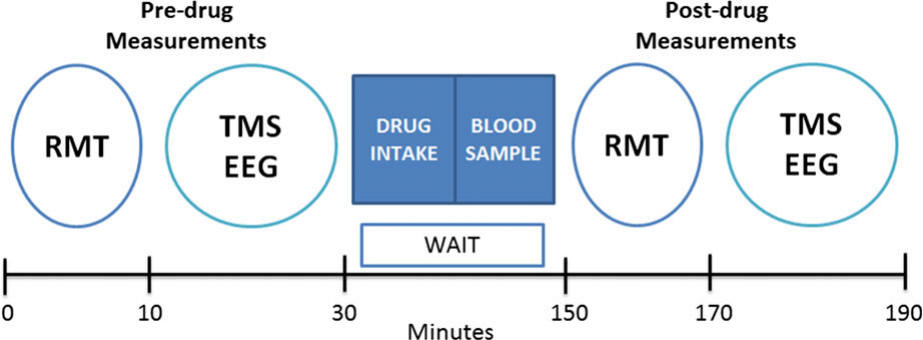
Experimental protocol and timeline. RMT and TMS‐EEG sessions were recorded before and 120 min after drug intake. Stimulation intensity during TMS‐EEG postdrug was set at 100% predrug RMT. Five minutes before postdrug measurement a blood sample was taken for each subject to verify drug plasma concentration.

A pseudo‐randomized, placebo‐controlled, double‐blinded crossover study was used to assess the acute effects of a single oral dose of the AEDs lamotrigine (300 mg) and levetiracetam (3,000 mg) on TEPs. These doses were used because they are effective standard daily doses for epilepsy treatment and in previous reports they have been effective in altering TMS measures of motor cortical excitability.^16^, ^17^, ^18^, ^19^ Lamotrigine is a voltage‐gated Na^+^ channel blocker, whereas levetiracetam acts primarily by binding to synaptic vesicle protein 2A (SV2A), modulating its actions to affect neuronal excitability and exerting seizure protection through effects on synaptic transmission.^1^ Subjects participated in three sessions spaced 1 week apart to exclude crossover effects and drug interference. The experimental protocol is described in Figure 1. First, RMT was measured predrug intake; next, TEPs were recorded before and 120 min after drug intake with 100% RMT predrug as intensity of stimulation. Drug‐induced changes of RMT were also measured. The timing of postdrug measurement was chosen according to drug pharmacokinetics and to previous studies reporting a significant alteration of motor cortical excitability at this time point.^16^, ^17^, ^18^, ^20^ A blood sample was taken 5 min before commencement of postdrug measurement to check drug plasma levels. Finally, 2 h after drug intake, subjects were asked to rate their level of sedation on an ordinal scale from 0 to 3, with 0 = no, 1 = mild, 2 = moderate, and 3 = severe sedation.^19^

### Transcranial magnetic stimulation (TMS) and electromyography (EMG)

Using a figure‐of‐eight coil (external diameter of each wing, 90 mm) connected to a Magstim 200^2^ magnetic stimulator (Magstim) with a monophasic current waveform, focal TMS of the hand area of the left primary motor cortex (M1) was performed. The optimal coil position to elicit motor evoked potentials (MEPs) in the first dorsal interosseous (FDI) muscle of the right hand was determined as the site where TMS consistently generated stable responses with amplitudes of ~1 mV. The hotspot position and the edge of each coil's wing were marked on top of the EEG cap using a felt tip pen. In addition, the same experimenter conducted all the TMS‐EEG sessions for each participant and study session. Coil position and orientation relative to the marked position were monitored carefully by the experimenter throughout stimulation and corrected if necessary (i.e., if the participant moved). MEP recordings were obtained by surface EMG using Ag‐AgCl cup electrodes in a belly‐tendon montage. The coil was held tangentially over the scalp with the handle pointing backward and about 45 degrees away from the midsagittal line. This orientation induces current flow in the lateral‐posterior to medial‐anterior direction, activating the corticospinal system transsynaptically via horizontal corticocortical connections, and is optimal for eliciting MEPs.^21^

Single TMS pulses were applied to determine the RMT using the relative frequency method.^22^ This was defined as the lowest stimulus intensity sufficient to elicit an MEP of >50 μV peak‐to‐peak amplitude in at least 5 of 10 trials while the FDI muscle was relaxed.

### Transcranial magnetic stimulation (TMS) and electroencephalography (EEG)

A TMS‐compatible EEG system was used to record TMS‐evoked EEG potentials (TEPs) (BrainAmp MRplus; Brain Products), which prevented EEG amplifier saturation and allowed continuous data recording throughout TMS. The EEG signal, digitized at a frequency of 5,000 Hz, was acquired continuously from 64 sintered Ag/AgCl electrodes (EasyCap 64Ch; Brain Products), with the impedance kept below 10 kΏ throughout the experiment. Subjects were on a comfortable reclined chair and asked to stay awake with their eyes open and focusing on a fixation cross, and they were asked to minimize eye blinks and other movements during the recording blocks. Earphones with a masking noise were used to avoid contamination of the EEG signal by auditory potentials induced by the click associated with the TMS coil sending pulses.^23^

Before and after drug intake, 150 pulses were delivered at an intensity of 100% RMT over the left M1 FDI hotspot, with a 4 s interstimulus interval and a variance of 20% to avoid adaptation (Fig. 1).

### TMS‐EEG data processing and analysis

After excluding trials with prominent eye movements, blinks, and muscle artifacts (on the basis of visual inspection), EEG data were analyzed using a MATLAB toolbox (FieldTrip, http://fieldtrip.fcdonders.nl/)^24^ following a multistep procedure.^7^ EEG data were downsampled to 1 kHz, segmented 1 s before and after the pulse, and a linear interpolation for ±10 msec was applied to remove the TMS artifact. Bad channels (mean number ± SD per subject 3 ± 0.5) were removed from the EEG, and the signal was reconstructed by interpolating the surrounding electrode signals. Data was then re‐referenced to the linked mastoids (TP9 and TP10 electrodes), notched filtered (50 Hz), and detrended. Residual artifacts related to the TMS pulse (e.g., TMS recharging artifact, muscle decay artifact), eye‐blinks, saccades, and muscle movement were removed by independent component analysis (FastICA).^25^ Components were removed on the basis of the spatiotemporal profile indicating the activation of temporal muscles, and by an activation of opposed polarity mainly recorded over frontocentral electrodes.^25^, ^26^, ^27^ Finally, remaining data were baseline corrected (from −600 to −100 ms) and a low pass filter was applied (45 Hz).

TEPs were calculated by averaging artifact‐free EEG trials (averaged number of trials across subjects before and after levetiracetam: 116 ± 14 and 98 ± 21; lamotrigine: 119 ± 14 and 104 ± 21; and placebo: 114 ± 21 and 111 ± 11). Specifically, five TEPs components (P = Positive, N = Negative) in accordance with the literature^7^, ^28^ were studied: P25 (time of interest, TOI [15–35 msec]), N45 (36–65 msec), P70 (65–90 msec), N100 (90–145 msec), and P180 (145–300 msec). TOIs were chosen on the basis of the grand‐averaged TEPs and kept identical during the analysis of predrug and postdrug measurements and across conditions. To analyze drug‐induced modulation of TMS‐evoked potentials, we selected a region of interest (ROI) that was composed of 12 channels over and around the stimulation site (left M1) and the corresponding contralateral site (FC1, FC3, FC5, C1, C3, C5, CP1, CP3, CP5, P5, P3, P1, FC2, FC4, FC6, C2, C4, C6, CP2, CP4, CP6, P2, P4, and P6).

### Statistics

To evaluate drug effects on RMT, a repeated‐measures analysis of variance (ANOVA) was performed with drug (three levels: levetiracetam, lamotrigine, placebo) and time (two levels: predrug, postdrug) as within‐subject factors.

To analyze significance of TEP amplitude modulations induced by lamotrigine and levetiracetam, multiple dependent sample *t*‐test comparisons (postdrug vs. predrug and between drug conditions) were applied for each TOI in all the electrodes within the indicated ROI. To correct for multiple comparisons (i.e., electrodes, time points), we conducted a nonparametric cluster‐based permutation analysis^29^ as implemented in FieldTrip. In particular, a paired *t*‐test was conducted for each electrode at each time bin within the specified TOIs. T‐values exceeding an a priori threshold of p < 0.05 were clustered based on adjacent time bins and neighboring electrodes. Cluster‐level statistics were calculated by taking the sum of the t‐values within every cluster. The statistical comparisons were done with respect to the maximum values of summed t‐values. By means of a permutation test (i.e., randomizing data across conditions and rerunning the statistical test 1,500 times), we obtained a reference distribution of the maximum of summed cluster t*‐*values to evaluate the statistic of the actual data. Clusters in the original dataset were considered to be significant at an alpha level of 5% if <5% of the permutations (N = 1,500) used to construct the reference distribution yielded a maximum cluster‐level statistic larger than the cluster‐level value observed in the original data.

Possible relations between the AED‐induced sedation, AED blood concentrations, and changes in TEPs were explored by applying Spearman correlation analysis.

## Results

All the subjects, except for one, tolerated the experimental protocol and the study medications. The common side effects were sedation, drowsiness, and dizziness, but these did not prevent the completion of the experiment. In ratings of sedation level for each condition (0 = no sedation, 1 = mild sedation, 2 = moderate sedation, and 3 = severe sedation), the average of all participants for the placebo was 0.4 ± 0.6 (n = 15), levetiracetam was 1.5 ± 0.9 (n = 15), and lamotrigine was 1.1 ± 0.8 (n = 14). A Friedman test indicated a significant effect of drug ($\chi_{2}^{2}$ = 11.4, p = 0.003). Averaged plasma concentration for levetiracetam was 57.4 ± 8.6 mg/L (reference range 6.0–20.0 mg/L) and for lamotrigine was 4.1 ± 0.8 mg/L (reference range 1.0–15.0 mg/L).

### Antiepileptic drug effects on TMS‐evoked EEG potentials

Repeated‐measures ANOVA of RMT data showed a significant main effect of time (F_1,14_ = 43.6, p < 0.001) and drug (F_2,28_ = 4.5, p = 0.02). In line with previous reports, there was a significant interaction between TIME and DRUG (F_2,28_ = 13.6, p < 0.001)*,* which was explained by a significant increase of RMT after intake of levetiracetam (p = 0.002) and lamotrigine (p < 0.001), whereas placebo had no effect on RMT (p > 0.05).^4^, ^18^ Finally, there were no significant differences between predrug RMT values, which were used as stimulation intensity for predrug and postdrug TMS‐EEG blocks (Fig. 1) (levetiracetam: 52.5 ± 6.7% maximum stimulator output [MSO]; lamotrigine: 51.7 ± 6.9% MSO; placebo: 51.8 ± 7.4% MSO; F_1,14_ = 0.65, p > 0.05).

**Figure 2:**
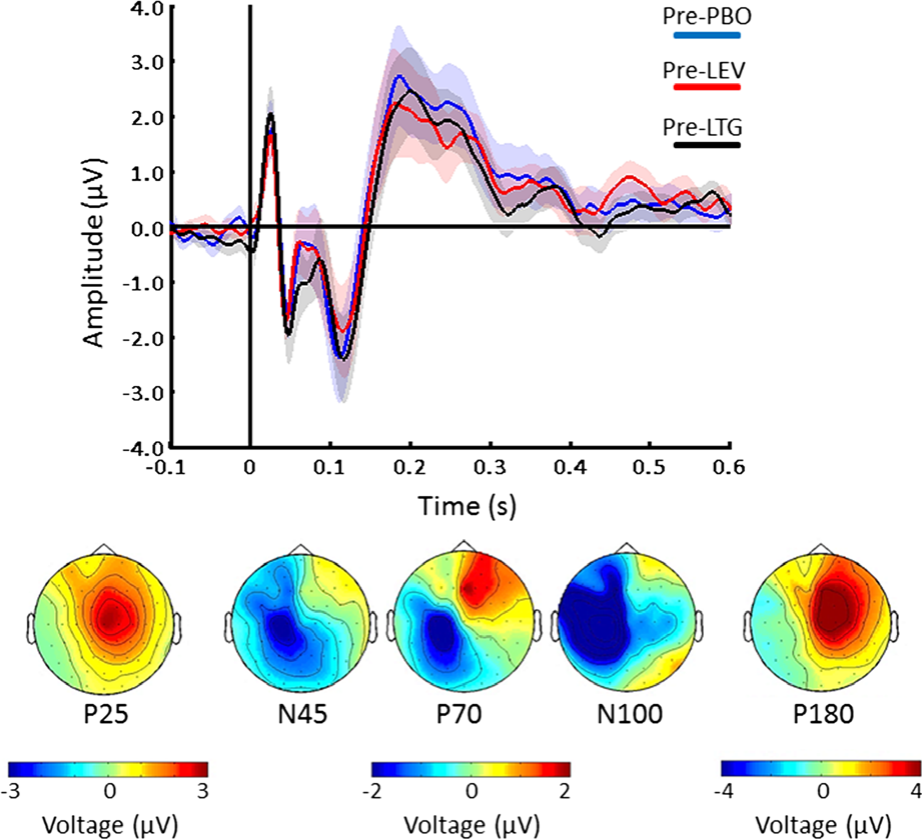
TEPs before drug intake. Grand‐averaged TEPs before intake of placebo (PBO, blue), levetiracetam (LEV, red), and lamotrigine (LTG, black). The vertical line at time 0 represents the time of the TMS stimulation. Bottom line shows topographic distribution of surface voltages for the most pronounced TEPs (P25, N45, P70, N100, and P180). Each plot shows the grand‐average across the three predrug measurements within the corresponding TOI and channels within ROI (shades indicate standard error of the mean [SEM]). For further details, see Materials and Methods.

The averaged EEG response after single‐pulse TMS of M1 at baseline showed typical TEP components (P25, N45, P70, N100, and P180) as previously found in literature.^7^, ^28^ The topography shows that P25 and P180 were positive and located respectively in central and contralateral site with respect to the left M1, whereas the N45, P70, and the N100 were located predominately in the ipsilateral hemisphere but spreading also over contralateral sites (Fig. 2). The cluster analysis did not show significant effects (p > 0.05) between the predrug conditions, showing high reproducibility across sessions.

**Figure 3:**
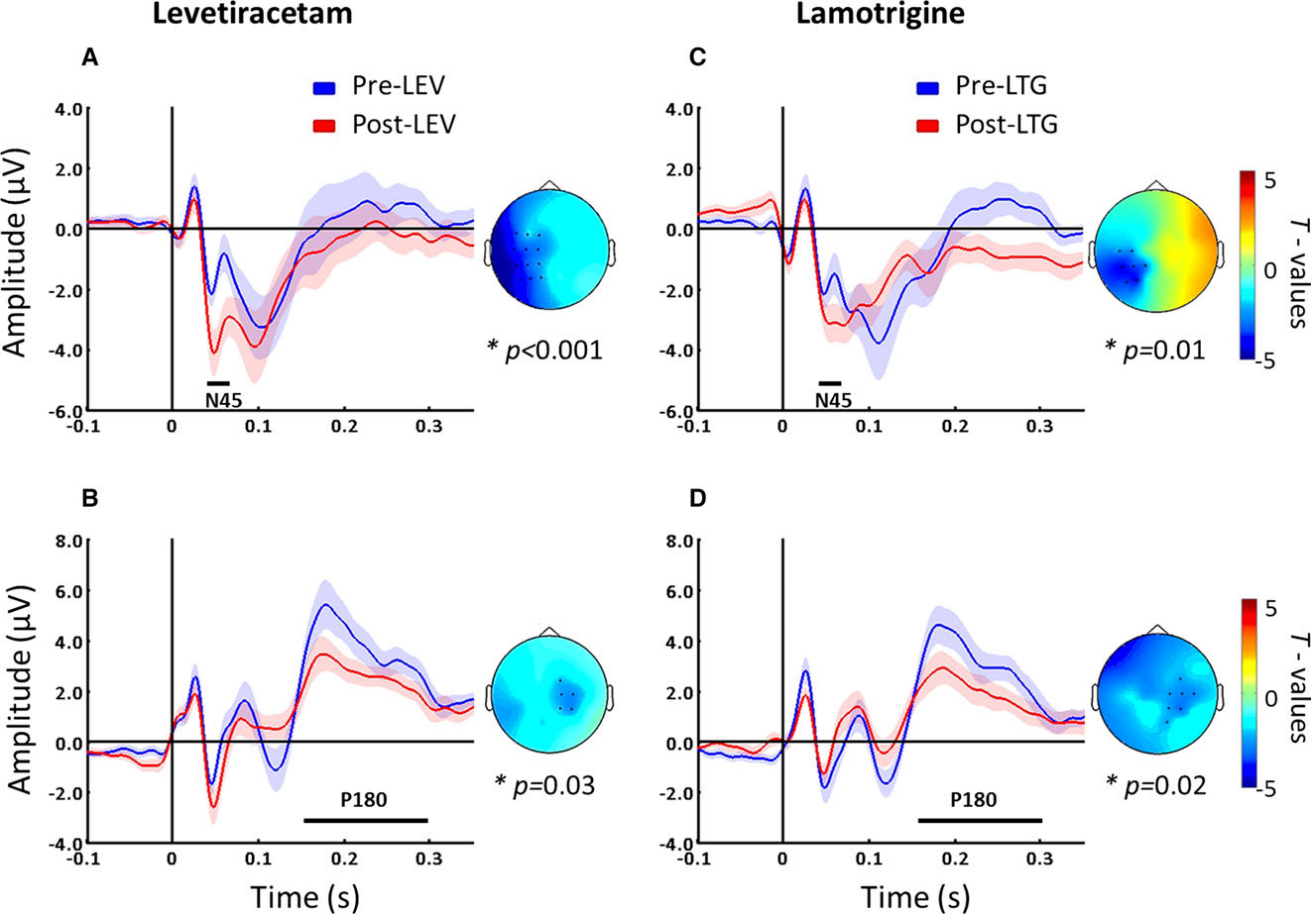
AED‐induced changes of TEPs, which were recorded before (blue) and after (red) intake of levetiracetam (LEV, left panel) and lamotrigine (LTG, right panel). Levetiracetam and lamotrigine increased the N45 potential ipsilaterally (**A**,** C**) and suppressed the P180 component contralaterally (**B**,** D**). Black bars underneath represent significant drug‐induced changes in TEPs. T‐statistic maps of the TEP amplitude showing postdrug versus predrug differences. Blue represents increase in negativity or reduced positivity (shades indicate SEM). Each plot shows the grand‐average across significant channels, which are indicated by black dots in the t‐statistic maps. For further details, see Materials and Methods.

The cluster‐based permutation analysis was applied between postdrug and predrug conditions to test the effect of AEDs on TEPs. Although placebo did not show any significant changes, levetiracetam and lamotrigine increased the amplitude of the N45 potential in channels close to the stimulation (pre‐levetiracetam: −2.73 ± 1.26 μV [n = 15]; post‐levetiracetam: −4.55 ± 2.56 μV [n = 15], p < 0.001, channels levetiracetam: FC1, FC3, FC5, C1, C3, C5, CP1, CP3, CP5, P1, P3, and P5; pre‐lamotrigine: −3.06 ± 1.51 μV [n = 14]; post‐lamotrigine: −4.00 ± 1.63 μV [n = 14], p = 0.01, channel lamotrigine: C3, C5, CP1, CP3, CP5, P3, and P5; Fig. 3A–C) and suppressed the P180 amplitude over channels contralateral to the stimulated area (pre‐levetiracetam: 6.26 ± 3.21 μV [n = 15]; post‐levetiracetam: 4.56 ± 1.82 μV [n = 15], p = 0.03, channel levetiracetam: FC2, C2, CP2, C4, and CP4; pre‐lamotrigine: 5.11 ± 2.45 μV [n = 14]; post‐lamotrigine: 3.80 ± 2.15 μV [n = 14]; and p = 0.02, channel lamotrigine: FC4, C2, C4, C6, CP2, CP4, and P2; Fig. 3B–D).

These results were confirmed by the comparison of post‐drug conditions. Compared to placebo, levetiracetam and lamotrigine increased the N45 component over channels ipsilateral to stimulation (levetiracetam channels: FC1, FC5, C1, C3, C5, and P3, p < 0.001; lamotrigine channels CP3, CP5, and P3, p = 0.01) and suppressed the P180 (levetiracetam channels: FC4, C4, and C6, p = 0.03; lamotrigine channels: C3, FC3, and FC5, p = 0.03). Furthermore, levetiracetam only suppressed the N100 potential over contralateral sites (channels: FC2, FC4, FC6, C6, and C4, p = 0.03). Finally the comparison between levetiracetam and lamotrigine did not show significant differences (p > 0.05 for all the TOIs). These results may suggest that these AEDs with different mechanisms of action could provoke similar modulation of TMS‐EEG activity.

**Figure 4:**
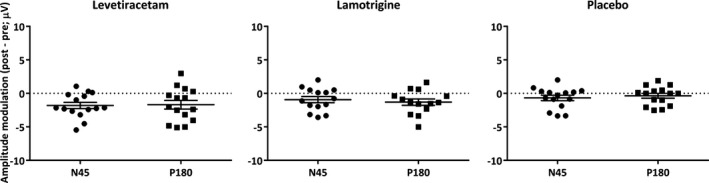
Single subject data of AED effects. The change in N45 and P180 amplitude (post‐pre drug, μV) has been extracted from individual subjects from the average of significant channels in the levetiracetam and lamotrigine conditions (cf. Fig. 3). For placebo, changes in the N45 and P180 were extracted from the average of channels within the ROI. Horizontal bars indicate group averages ± SEM.

We investigated the AED‐induced changes of the N45 and P180 components at the level of individual subjects. Although most of the subjects revealed an enhancement of the N45 and suppression of the P180 potentials after levetiracetam and lamotrigine intake, a few participants showed opposite trend (Fig. 4).

### Correlations

We explored possible relations between AED‐induced modulation of TEPs and drug plasma concentration as well as drug‐induced level of sedation. The correlation analyses were not significant (p > 0.05), suggesting that TEP changes are not related to the amount of drug in the blood or to sedation.

## Discussion

We utilized TMS‐EEG to obtain cortical signatures of two widely used AEDs in healthy participants. Despite the different molecular targets, we found that both lamotrigine and levetiracetam modulated TEPs in a similar trend. At group level, they significantly increased the N45 potential and decreased the amplitude of later components. These results are reflected at the individual level; however, a few subjects showed opposite effects compared to the group on average (Fig. 4). Levetiracetam is a broad spectrum AED used to treat focal and generalized epilepsy and has a unique mechanism of action involving binding to SV2A vesicles, which are widely distributed among synapses. The synaptic vesicle protein 2 (SV2) help transportation of common constituents of the vesicles, for example, calcium or adenosine triphosphate, and controls exocytosis of vesicular neurotransmitters.^30^ Thus levetiracetam reduces neuronal excitability and synaptic transmission of excitatory neurotransmitters to prevent epileptic hyperexcitability.

Lamotrigine also is a broad spectrum AED used in both generalized and focal epilepsy, and it exerts its anticonvulsant effect via blockade of presynaptic voltage‐gated sodium channels to stabilize the presynaptic neuronal membrane and thus reduce neurotransmitter release.^31^

### Effects of levetiracetam and lamotrigine on N45

It has been demonstrated that the N45 component reflects fast inhibitory postsynaptic inhibition mediated by GABA_A_ receptors, as positive allosteric modulators (i.e., benzodiazepines) increased the N45 amplitude.^7^ Previous research on in vitro and in vivo models has suggested that levetiracetam does not act directly on GABA_A_ receptors but works via indirect mechanisms to increase GABAergic activity.^32^ Previous studies have also found that the N45 was augmented only in areas contralateral to the stimulation site with direct GABA_A_ positive modulators.^7^ However in the current study, the effect of levetiracetam on the topography of N45 shows a widespread negativity, suggesting that N45 is modulated by widespread GABA_A_ergic neuronal pathways.

Lamotrigine does not act primarily on GABA_A_ receptors, and conflicting results have been reported about the activity of lamotrigine on GABA and GABA_A_ receptors. For instance, lamotrigine reduced GABA_A_ receptor–mediated synaptic transmission in the basolateral amygdala of rats, a brain area relevant for epileptogenesis and affective disorders.^33^ Braga et al. speculated that lamotrigine acts at GABAergic presynaptic terminals to reduce GABA release by decreasing calcium influx into GABAergic terminals, either by directly modulating voltage‐gated calcium channels or by indirectly by inhibiting sodium channels. However in experiments on rat entorhinal cortex, lamotrigine enhanced the amplitude and frequency of spontaneous GABA_A_ receptor–mediated inhibitory postsynaptic currents by increasing GABA release.^31^ This inconsistency in observations could be due to lamotrigine action on different brain areas. More recently it has been suggested that lamotrigine does exert a GABAergic mechanism, as it potentiated protective effects of GABA_A_ receptor agonists in a model of Huntington's disease; the authors suggested it could be acting by modulation of GABA‐binding sites.^34^

### Effects of levetiracetam and lamotrigine on late latency components

In this study, the P180 waveform was significantly lower after the consumption of both AEDs; in addition levetiracetam, compared to placebo, suppressed the N100 component. Generally the P180 peak has been associated with auditory evoked activity generated by the coil click during stimulation.^35^ However, this possible cofound can be controlled by applying a masking sound throughout the TMS session.^23^ Moreover, the postdrug measurements have been obtained with the same stimulation intensity as predrug measurements (100% RMT at predrug intake); therefore, we exclude a connection with auditory effects.

The suppression of the N100/P180 waveform complex has been a key finding in a previous study that involved patients with epilepsy.^9^ Results have been interpreted as a decreased inhibitory state in patients. Of interest, all patients were treated with levetiracetam; therefore, given our present findings, it is likely that the reduction of the N100/P180 complex may be linked to levetiracetam‐induced modulation.

A large body of evidence showed that the N100 potential is linked to GABA_B_ receptor–mediated neurotransmission. Notably, the N100 amplitude increased after the administration of the GABA_B_ agonist baclofen, and it correlated with the duration of the cortical silent period, a TMS‐EMG marker of GABA_B_ receptor activity.^7^, ^8^, ^36^ Therefore, a suppression of this waveform may indicate decreased GABA_B_ receptor–mediated inhibition. However, this finding is in contrast with a TMS‐EMG study that showed that levetiracetam prolonged the cortical silent period (CSP).^18^ Although it is well known that levetiracetam and lamotrigine increased RMT, their indirect activity on GABAergic inhibitory circuits still needs to be fully elucidated. Analysis of event‐related spectral perturbations (ERSPs) may provide additional knowledge and novel candidate biomarkers to be validated in patients.

On average, participants experienced the highest level of sedation/fatigue with levetiracetam and the second highest with lamotrigine; this concurs with previous work, which found that drugs acting on GABA have the highest rate of fatigue, followed by levetiracetam, and that those acting on sodium channels have the lowest incidence of fatigue, that is, lamotrigine.^37^ These results are also in accordance with the blood plasma concentrations of the drugs; levetiracetam had the highest average concentration in blood outside the reference range, with lamotrigine averaging toward a lower concentration for its reference range. Finally, the AEDs present in the blood and their induced sedation were independent from the observed modulation of TMS‐EEG metrics. Furthermore, AED‐induced changes of TEPs did not correlate with their serum level. Hence, in agreement with previous studies, the present results indicate that TMS‐EEG has the potential to provide additional useful information, compared to the AED blood level, about effects of drugs on cortical excitability.^17^, ^38^, ^39^ Future studies should investigate the relation between these TMS‐EEG candidate biomarkers and the antiepileptic activity of lamotrigine and levetiracetam. If results turn out to be successful, TMS‐EEG may develop a role as an early marker of long‐term treatment outcome. In addition, it could be integrated in early phase screening of newly developed AEDs.

Our results showed that, despite the varying profile of effects and regardless of the (putative) molecular targets of the different drugs, systemically administered lamotrigine and levetiracetam exert similar modulation of TEPs. Similar results were obtained in studies in vitro, which showed that different AEDs, irrespective of their molecular mechanisms, can increase the excitation/inhibition balance and reduce overall neuronal and network excitability in the rat entorhinal cortex.^40^

In conclusion, TMS‐EEG may identify that different AEDs have a common pathway of therapeutic effect that involves increased neuronal inhibition, irrespective of the primary molecular target.

## Disclosure

None of the authors has any conflict of interest to disclose. We confirm that we have read the Journal's position on issues involved in ethical publication and affirm that this report is consistent with those guidelines.
